# The Impact of Outdoor Physical Activities on the Health of Pregnant Women: A Scoping Review

**DOI:** 10.3390/healthcare14091211

**Published:** 2026-04-30

**Authors:** Nikola Stojanović, Milica Filipović, Vladimir Miletić, Biljana Vitošević, Tijana Purenović-Ivanović, Željko Rajković, Jovana Vitošević, Katarina Milanović, Slavka Durlević, Igor Ilić

**Affiliations:** 1 Faculty of Sport and Physical Education, University of Niš, 18000 Niš, Serbia; tijanapurenovic@gmail.com; 2 Faculty of Sport and Physical Education, University of Priština in Kosovska Mitrovica, 38218 Leposavić, Serbia; biljana.vitosevic@pr.ac.rs (B.V.); igor.ilic@pr.ac.rs (I.I.); 3 Faculty of Sport and Physical Education, University of Belgrade, 11000 Belgrade, Serbia; vladimir.miletic@fsfv.bg.ac.rs (V.M.); zeljko.rajkovic@fsfv.bg.ac.rs (Ž.R.);; 4 Department of Business Studies, Toplica Academy of Applied Studies (TAAS), 18420 Blace, Serbia; jovana.vitosevic@vpskp.edu.rs; 5 Faculty of Sport and Physical Education, University of Novi Sad, 21000 Novi Sad, Serbia; durlevicslavka3@gmail.com

**Keywords:** pregnancy, outdoor physical activity, green exercise, gestational diabetes, mental health

## Abstract

**Background**: Outdoor physical activity encompassing structured exercise, recreational activities, and environmentally facilitated movement, during pregnancy may confer benefits through both exercise and exposure to natural environments and sunlight. However, evidence on maternal health outcomes remains heterogeneous and has not been systematically mapped. This scoping review aimed to synthesize research on outdoor physical activity and nature-based environmental exposure during pregnancy, and their associations with maternal metabolic, cardiovascular, and mental health outcomes, as well as pregnancy-related outcomes. **Methods**: The review followed PRISMA-ScR guidelines. PubMed, Web of Science, Scopus, and Google Scholar were searched for studies published between January 2013 and February 2026. Records were screened against predefined eligibility criteria. Methodological quality was assessed with design-specific tools: PEDro for randomized controlled trials; the Newcastle–Ottawa Scale for cohort and case–control studies; and JBI and CASP checklists for cross-sectional, quasi-experimental, pilot, and qualitative studies. **Results**: Of 935 identified records, 22 met the eligibility criteria. Outdoor exposure was operationalized through structured outdoor programs (mainly walking-based), self-reported outdoor activity patterns, and environmental indicators such as residential greenness and neighborhood walkability. Outcomes clustered into metabolic and cardiometabolic measures, mental health indicators, pregnancy and birth outcomes, and behavioral or environmental determinants of physical activity. Overall, most studies (17 of 22) reported at least one positive association, with the most consistent evidence for metabolic outcomes and mental health, whereas pregnancy and birth outcomes yielded more heterogeneous results. **Conclusions**: Outdoor physical activity and supportive natural or built environments may be associated with favorable maternal metabolic and mental health during pregnancy. Nevertheless, heterogeneity in exposure assessment and study design limits comparability and constrains causal inference. Future research should standardize outdoor exposure metrics and apply designs capable of isolating environmental effects from exercise alone.

## 1. Introduction

Physical activity (PA) plays a central role in maintaining overall health and reducing the risk of chronic noncommunicable diseases across the lifespan [1]. During pregnancy, regular physical activity is particularly important, as women experience substantial physiological and psychological changes that may increase vulnerability to adverse outcomes [2,3]. These include an elevated risk of gestational diabetes mellitus (GDM), preeclampsia, excessive gestational weight gain, and heightened stress and mood disturbances [2,4]. Accordingly, maintaining adequate levels of physical activity during pregnancy is increasingly recognized not only as a strategy for general health promotion but also as a targeted approach to mitigate pregnancy-specific metabolic and cardiovascular risks [5,6,7]. Traditionally, exercise recommendations for pregnant women have emphasized activities performed in controlled or indoor settings, often prioritizing structured programs designed to minimize perceived environmental risks [8]. However, a growing body of evidence suggests that outdoor physical activity may provide additional benefits beyond those attributable to exercise alone, potentially through the combined effects of movement, exposure to natural environments, and sunlight [9].

Current international guidelines consistently endorse regular physical activity during pregnancy. The World Health Organization (WHO) recommends that pregnant women without medical contraindications engage in at least 150 min of moderate-intensity aerobic physical activity per week [10]. The appropriate intensity of physical activity during pregnancy depends on the individual’s pre-pregnancy activity level and clinical status [11]. Previously sedentary women are advised to begin with low-intensity activities and increase duration and effort gradually, whereas women who were habitually active before conception may safely continue vigorous-intensity exercise throughout pregnancy, provided that adequate caloric intake is maintained and prolonged exertion beyond 45 min is managed to minimize the risk of hypoglycemia [8]. For the majority of pregnant women, moderate-intensity aerobic activity, defined as exercise that raises heart rate while still permitting conversation and corresponding to a perceived exertion of 13–14 on the Borg scale, represents the most widely endorsed target; commonly recommended forms include brisk walking, stationary cycling, swimming, dancing, and water aerobics [8]. These recommendations include activities that may be performed outdoors, provided that environmental conditions, safety considerations, and individual health status are appropriately assessed [12]. Most national guidelines advocate moderate-intensity activities such as walking, swimming, and stationary cycling, along with pelvic floor and light resistance exercises [13]. Although these guidelines do not specify whether activities should be performed indoors or outdoors [10,13], several of the most frequently recommended activities, particularly walking, are inherently adaptable to natural and neighborhood environments. This creates a conceptual and practical overlap between structured exercise recommendations and exposure to outdoor settings, suggesting that a substantial proportion of recommended pregnancy-safe activity may simultaneously involve environmental exposure with potential additive or synergistic health effects [9,14].

Observational evidence indicates that physical activity during pregnancy may be associated with higher serum 25-hydroxyvitamin D concentrations, even after accounting for seasonal variation and individual characteristics [15,16]. This association is particularly relevant in the context of outdoor physical activity, where sun exposure may independently contribute to vitamin D status beyond the metabolic effects of exercise [17]. In addition to sunlight-related pathways, environmental health research suggests that exposure to green spaces may support mental health through stress-reduction, attentional restoration, and affect-regulation mechanisms, while also indirectly promoting physical activity engagement [18]. These dual pathways, behavioral activation and environmental exposure, may help explain associations observed between outdoor activity and maternal health outcomes during pregnancy [19]. Furthermore, natural environments have been linked to psychological benefits, including reduced perceived stress, improved mood, and enhanced emotional well-being [14,20]. Moderate outdoor activities such as walking in parks or light hiking may also improve cardiovascular function and have been suggested to reduce the risk of hypertensive disorders of pregnancy, including preeclampsia [21]. Together, this body of evidence suggests that outdoor physical activity during pregnancy may operate through both physiological and psychosocial pathways, underscoring the importance of distinguishing outdoor activity from indoor exercise.

Despite established recommendations and documented benefits, several studies report a marked decline in physical activity levels as pregnancy progresses [2,22,23,24,25]. Population-based data indicate that many women reduce their activity shortly after conception, with this downward trajectory continuing throughout gestation [2,22]. Estimates suggest that only around 20% of pregnant women participate in structured exercise programs, while nearly half discontinue their pre-pregnancy activity routines [22]. Adherence to recommended guidelines declines from approximately 40% in early pregnancy to about 27% in the late third trimester [23]. Objective accelerometer-based data further confirm a substantial reduction in moderate-to-vigorous physical activity during late pregnancy, often extending into the postpartum period [24]. These pre-existing downward trends were further accentuated during the COVID-19 pandemic, when movement restrictions and safety concerns led to additional reductions in physical activity among pregnant women [25]. Emerging evidence linked these behavioral changes to unfavorable metabolic outcomes, such as increased gestational diabetes incidence [26], while outdoor physical activity was identified as a coping strategy associated with lower anxiety and depressive symptoms [27]. These observations further underscore how access to outdoor environments may shape maternal health trajectories.

In addition to physiological barriers inherent to pregnancy, multiple social, environmental, and infrastructural factors appear to influence women’s participation in outdoor physical activity [28,29]. Hormonal changes, fatigue, and pregnancy-related discomfort may reduce activity levels, while external constraints such as limited access to safe walking paths or green spaces, adverse weather conditions, safety concerns, and cultural norms may further shape engagement [30,31]. Qualitative and observational studies consistently identify perceived safety, social support, and environmental accessibility as important correlates of continued participation in outdoor physical activity during pregnancy [28,32]. Together, these findings might suggest that engagement in outdoor activity is influenced not only by individual health status but also by characteristics of the surrounding built and natural environment [28,31,32].

For the purposes of this review, outdoor physical activity is defined as any form of physical activity performed in outdoor settings, including structured exercise in natural environments [14], habitual recreational activities such as walking and cycling, and physical activity facilitated by environmental characteristics such as residential greenness and neighborhood walkability [18].

Despite this growing body of evidence, no previous review has systematically mapped how outdoor physical activity during pregnancy has been conceptualized, measured, and linked to maternal health outcomes across different study designs. The absence of a comprehensive synthesis limits the ability to identify consistencies in findings, compare exposure definitions, and guide future research priorities. Given the growing, yet heterogeneous, body of literature on outdoor physical activity and environmental exposure during pregnancy, a scoping review was selected as the most appropriate methodological approach. Scoping reviews are designed to map the extent, range, and nature of evidence within emerging or conceptually broad fields, particularly when study designs, exposure definitions, and outcome measures vary substantially [33,34]. This approach is especially relevant in the post-pandemic context, where shifts in mobility patterns and environmental use may have altered patterns of outdoor engagement [35]. Accordingly, the aim of this review is to map and synthesize existing research on outdoor physical activity and nature-based environmental exposure during pregnancy and their relationships with maternal metabolic, cardiovascular, and mental health outcomes, as well as pregnancy-related outcomes. The specific research questions are: (1) What associations have been reported between outdoor physical activity and metabolic and mental health outcomes during pregnancy? (2) What motivational factors and barriers have been identified in relation to continued participation in outdoor physical activity? The review focuses on the literature published since 2013, a period marked by growing research attention to outdoor activity and exposure to green spaces in maternal health contexts.

## 2. Materials and Methods

The protocol for this scoping review was registered on the International Platform of Registered Systematic Review and Meta-analysis Protocols (INPLASY202620061) to enhance transparency and methodological clarity. The review was conducted and reported in accordance with the PRISMA-ScR (Preferred Reporting Items for Systematic Reviews and Meta-Analyses extension for Scoping Reviews) guidelines.

### 2.1. Search Strategy

A comprehensive electronic search was conducted to identify studies examining outdoor physical activity and nature-based environmental exposure in relation to maternal health outcomes during pregnancy. The search strategy combined controlled vocabulary terms, including Medical Subject Headings in PubMed, with free text keywords related to pregnancy, physical activity, exposure to outdoor or green and blue spaces, and maternal health outcomes. The following electronic databases were systematically searched: PubMed, Scopus, and Web of Science Core Collection. Searches were limited to studies published between January 2013 and February 2026. Search strategies were adapted to each database’s indexing structure while maintaining conceptual consistency across platforms. The search was designed and executed by two reviewers (MF and SD), and the final search was conducted on 10 February 2026. The search strategy was independently verified by a third reviewer (II) to ensure completeness and accuracy. The temporal boundary of January 2013 was established on empirical grounds. Prior to 2012, research on physical activity during pregnancy rarely distinguished between indoor and outdoor settings. The first study to examine associations between green space exposure and pregnancy outcomes appeared in 2012 [36], and the first prospective cohort study to explicitly assess outdoor physical activity during pregnancy was published in 2013 [37]. By selecting January 2013 as the start date, the review captures the period during which outdoor exposure began to be treated as a distinct variable in perinatal research. The complete search strings for each database are presented in Table 1.

In addition to the database searches, Google Scholar was used as a supplementary source to identify potentially relevant studies not captured through database indexing. Due to the high volume of records, results were screened by relevance, and the first 300 records were reviewed. Eligibility criteria included peer-reviewed English-language articles involving human participants. Reference lists of all included full-text articles were manually screened to identify additional relevant studies.

### 2.2. Study Selection and Screening Process

Study selection was conducted in accordance with the PRISMA ScR guidelines. Records retrieved from PubMed, Web of Science Core Collection, and Scopus were exported into Zotero (version 7.0.30) for reference management. Duplicate records were identified using Zotero’s duplicate-detection function, then manually reviewed to confirm true duplicates and identify records with minor bibliographic differences across databases. Matching and verification were based on available citation metadata, including title, authors, year, journal, and DOI where available. This process removed 209 duplicate records from the database searches, leaving 426 unique records. Google Scholar results (*n* = 300) were screened separately due to the high volume and limited export compatibility and were reviewed for relevance independently by the same reviewers. Titles and abstracts of all remaining records were screened independently by two reviewers (SD and MF) according to predefined eligibility criteria. Potentially relevant articles were obtained in full text and assessed independently by the same reviewers to determine final eligibility. Disagreements at any stage were resolved through discussion and consensus, with consultation of a third reviewer (II) when necessary. The search strategy and eligibility criteria were developed collaboratively by the methodology team (MF, T.P.-I., and II). Data curation was performed by NS and JV, and validation of extracted data was conducted by NS, VM, and ŽR.

### 2.3. Data Charting Process and Data Items

Data charting was conducted using a structured extraction form developed by the review team in Microsoft Word (Microsoft Corp., Redmond, WA, USA). The form was pilot-tested and refined iteratively during the extraction phase to ensure clarity, completeness, and consistency in capturing study characteristics and exposure definitions. One reviewer (MF) performed the initial data charting for all included studies. A second reviewer (NS) independently verified the extracted data against the original full texts using the same framework. Discrepancies were resolved through discussion and consensus during joint review meetings. Particular attention was given to the operationalisation of outdoor exposure, especially in cases where the setting of physical activity was not explicitly reported. When information was unclear or not reported in the original study, it was recorded as missing and was not inferred by the review team.

The following data items were charted: author, year, and country; study design; sample characteristics (including sample size, gestational age or trimester at assessment, and clinical profile where applicable); definition of outdoor exposure (structured outdoor activity programme, self-reported outdoor activity, or environmental proxy measures such as residential greenness, NDVI, neighbourhood walkability, or high-altitude exposure); exposure or intervention characteristics (type, duration, frequency, and intensity or step targets when reported); comparator (if applicable); outcome domains and measurement approaches (metabolic, cardiovascular or hypertensive, mental health, pregnancy or birth, and behavioural, social, or environmental determinants); and key findings relevant to the review questions.

### 2.4. Eligibility Criteria

Eligibility criteria were defined a priori using the PICOS framework and are summarised in Table 2. Studies were eligible if they included pregnant women at any gestational age and examined outdoor physical activity or physical activity in natural, green, or blue spaces. Eligible studies were required to report at least one physical or mental health outcome during pregnancy, including metabolic outcomes (e.g., gestational diabetes, glycaemic control, gestational weight gain), cardiovascular or hypertensive outcomes (e.g., preeclampsia), mental health outcomes (e.g., stress, anxiety, depression), or pregnancy and birth outcomes (e.g., preterm birth, mode of delivery, macrosomia). All original peer-reviewed research designs were considered eligible, including randomized controlled trials, cohort studies, case–control studies, cross-sectional studies, quasi-experimental studies, pilot studies, case reports or case series, and qualitative studies. Only studies published in English between 2013 and 2026 were included. Studies were excluded if they focused exclusively on indoor physical activity, did not report health-related outcomes during pregnancy, or were not original research (e.g., systematic reviews, scoping reviews, meta-analyses, conference abstracts without full text, editorials, commentaries, guidelines, study protocols, theses, dissertations, or other grey literature).

### 2.5. Quality Assessment

Although critical appraisal is not mandatory for scoping reviews, methodological quality was assessed to provide contextual insight into the robustness and limitations of the included evidence. Quality appraisal was conducted using validated tools appropriate to each study design. Two reviewers (SD and MF) independently performed the assessments. Discrepancies were resolved through discussion and consensus, with consultation of a third reviewer (II) when required. Validation of the quality appraisal results was performed by NS, VM, and ŽR. Randomized controlled trials (*n* = 5) were evaluated using the PEDro Scale. Cohort and longitudinal observational studies (*n* = 4) and the case–control study (*n* = 1) were assessed using the Newcastle–Ottawa Scale (NOS). Cross-sectional studies (*n* = 6) were appraised using the Joanna Briggs Institute (JBI) Critical Appraisal Checklist for Analytical Cross-Sectional Studies, while quasi-experimental studies (*n* = 2) were evaluated using the corresponding JBI checklist. Qualitative studies (*n* = 2) were assessed using the CASP Qualitative Checklist. The pilot trial (*n* = 1) and the case report (*n* = 1) were appraised using the most appropriate JBI critical appraisal tool for their respective designs. Studies were classified as high or moderate methodological quality according to the scoring guidance provided within each appraisal instrument. No studies were excluded on the basis of quality assessment. Detailed appraisal results are presented in Table 3.

## 3. Results

**Figure 1:**
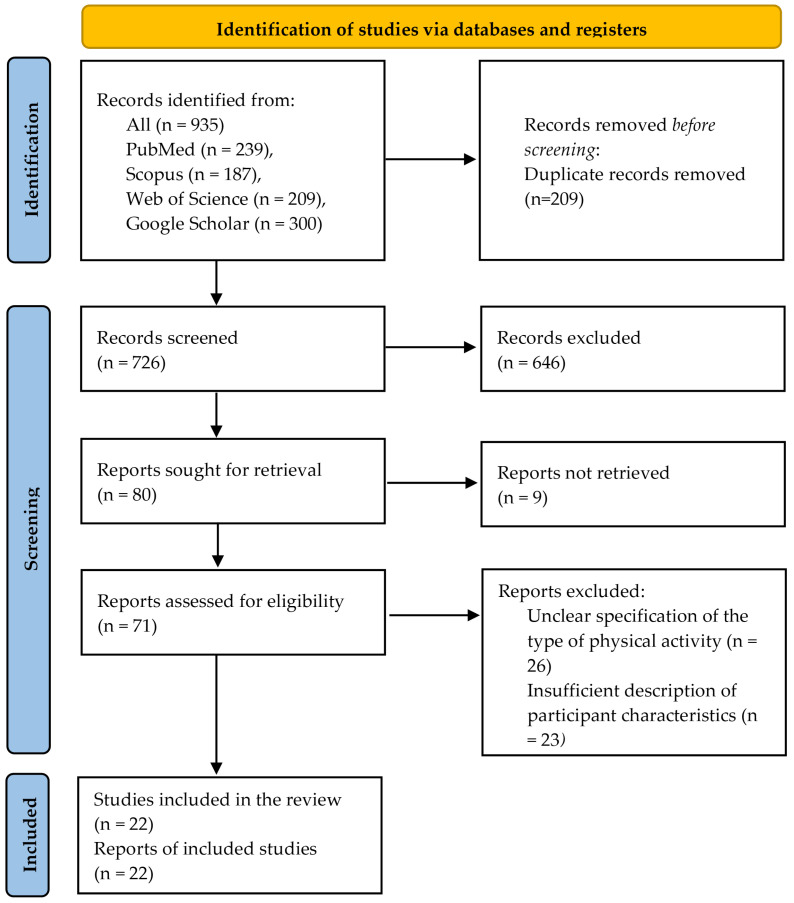
PRISMA-ScR flow diagram of study selection.

A total of 935 records were identified through database and supplementary searches (PubMed: *n* = 239; Scopus: *n* = 187; Web of Science: *n* = 209; Google Scholar: *n* = 300). After removing 209 duplicate records from database searches, 726 records underwent title and abstract screening. Of these, 646 were excluded for failing to meet the predefined eligibility criteria. Eighty full-text articles were sought for retrieval, of which nine were not retrieved. Seventy-one full-text articles were assessed for eligibility. Of these, 49 studies were excluded due to insufficient specifications of outdoor physical activity (*n* = 26) or inadequate reporting of participant characteristics (*n* = 23). Ultimately, 22 studies met all eligibility criteria and were included in the final scoping review. The study selection process is presented in the PRISMA flow diagram (Figure 1).

The following sections describe the characteristics of the included studies and summarise the findings across outcome domains. The included evidence base illustrates substantial heterogeneity in how outdoor exposure was conceptualized, operationalized, and measured across geographical contexts and methodological designs.

Most studies were conducted in Europe, including Denmark, Italy, the United Kingdom, Poland, Slovenia, and one Italy/Ukraine collaboration [28,32,37,38,39,41,51,53]. Additional studies originated from North America, primarily the United States and Canada [3,27,40,50,52], as well as from Asia and the Middle East (India, Japan, China, Iran, Egypt, and Ghana) [42,45,46,47,48,49,54]. Evidence from Oceania was represented by New Zealand [32], and one case report was conducted in Nepal in a high-altitude context [44].

Study designs spanned randomized controlled trials [38,39,46,49,50], quasi-experimental and pilot interventions [3,42,47], observational cohort, cross-sectional, and case–control studies [27,37,40,41,43,48,51,52,53,54], qualitative studies [28,32], and one case report [44]. Sample sizes ranged from a single participant in an extreme-environment case report [44] to a population-based dataset of approximately 3.28 million singleton births [52]. Several observational cohorts and cross-sectional datasets included several thousand participants [37,40,43,45], whereas intervention studies typically enrolled between several dozen and a few hundred participants [38,39,46,47,49,50].

Gestational timing varied substantially. Many studies recruited participants during the second trimester [3,28,37,38,39,41,42,46,47], while others examined exposure across multiple trimesters from early pregnancy to delivery [43,50]. A smaller subset focused specifically on late pregnancy [44,49], and several large observational analyses captured exposure patterns throughout gestation or around delivery [27,40,42,52,54].

To facilitate synthesis, the included studies were classified according to how outdoor exposure was operationalized: (a) structured walking-based interventions with defined duration and frequency parameters (*n* = 8); (b) self-reported habitual outdoor activity (*n* = 5); and (c) environmental proxy measures such as residential greenness and neighborhood walkability (*n* = 4). The remaining five studies focused on specific contexts, including cycling as a commuting mode, high-altitude exposure, comparisons between yoga and walking, and a qualitative exploration of barriers to outdoor activity. This classification informed the interpretive synthesis presented below and in the Discussion. Outcomes clustered into five domains: metabolic and cardiometabolic measures; cardiovascular and hypertensive-related outcomes; mental health indicators; pregnancy and birth outcomes; and behavioral or environmental determinants of physical activity.

### 3.1. Associations with Maternal Health Outcomes

#### 3.1.1. Metabolic and Cardiometabolic Outcomes

Seven studies examined metabolic or cardiometabolic outcomes, including serum vitamin D status, glycaemic control, gestational diabetes indicators, and gestational weight gain [37,38,42,45,47,50,52]. Multiple walking-based interventions and observational studies reported associations between outdoor activity and more favorable metabolic profiles, including higher vitamin D concentrations and improved glucose-related measures [37,38,42,50,52]. However, findings related to gestational weight gain and broader birth outcomes were mixed; for example, a combined nutrition and exercise intervention reported improvements in dietary intake without significant effects on gestational weight gain or birth outcomes [50]. Across exposure categories, the most consistent metabolic findings emerged from structured walking interventions targeting women with gestational diabetes or elevated metabolic risk, whereas studies using environmental proxy measures linked walkability to lower gestational diabetes rates at the population level without directly assessing individual metabolic response [52].

#### 3.1.2. Cardiovascular and Hypertensive Outcomes

Three studies examined cardiovascular and hypertensive-related outcomes, including maternal blood pressure, hypertensive disorders of pregnancy, and autonomic stress responses [39,46,53]. Walking-based interventions were associated with reductions in maternal blood pressure and lower incidence of hypertensive outcomes in two trials [39,46]. One activity-modality comparison showed differential autonomic responses, with higher parasympathetic activity following yoga than moderate-intensity walking [53]. All three studies in this domain employed structured intervention designs; no study using self-reported activity or environmental proxy measures reported cardiovascular outcomes, limiting the scope of cardiovascular evidence to a single exposure category.

#### 3.1.3. Mental Health Outcomes

Six studies examined mental health outcomes, most commonly stress, anxiety, and depressive symptoms, through both environmental exposure indicators and behavioral measures [3,27,28,32,41,54]. Several observational studies reported associations between greater green space exposure or outdoor engagement and lower symptom levels [27,41,54]. Qualitative evidence highlighted perceived psychological benefits of outdoor activity while also identifying barriers such as safety concerns, sociocultural constraints, and environmental limitations [28,32]. At the same time, not all findings were consistent; one cohort study reported no association between green space exposure and meeting physical activity recommendations after adjustment, alongside an overall decline in physical activity across pregnancy [51]. In contrast to the metabolic and cardiovascular domains, positive associations with mental health were reported across all three exposure categories—structured interventions [3], self-reported outdoor activity [27,32], and environmental proxy measures [41,54], suggesting that both active engagement and passive environmental exposure may be relevant to maternal psychological well-being.

#### 3.1.4. Pregnancy and Birth Outcomes

Seven studies addressed pregnancy and birth outcomes, including preterm birth, labor progression indicators, induction and cesarean delivery rates, and broader perinatal outcomes [38,40,48,49,50,51,52]. One case–control study reported a U-shaped association between outdoor activity duration and preterm birth risk [48]. A late-pregnancy walking intervention was associated with improvements in labor indicators and a reduced need for operative delivery, without adverse neonatal effects [49]. Population-based analyses examined associations between walkability and pregnancy behaviors and outcomes [52]. High-altitude exposure studies reported generally low complication rates overall but also indicated potential elevations in selected perinatal risks, whereas a single case report described no apparent maternal, fetal, or neonatal complications during sustained high-altitude activity [40,44]. Pregnancy and birth outcomes yielded the most heterogeneous results across the included studies, with findings varying by exposure type, gestational timing of assessment, and outcome definition. Both protective dose-response patterns [48] and null findings [50] were reported within the same outcome domain.

### 3.2. Determinants, Barriers, and Facilitators of Outdoor Physical Activity

Several studies provided evidence on factors influencing participation in outdoor physical activity during pregnancy, including motivational factors, barriers, and environmental determinants. Two qualitative studies identified perceived safety, social support, cultural norms, and environmental accessibility as key determinants of outdoor activity engagement among pregnant women [28,32]. One prospective cohort reported that neighborhood green space was not independently associated with meeting physical activity recommendations after adjustment for residential self-selection [43], suggesting that environmental availability alone may not be sufficient to promote activity. A cross-sectional study found that physical activity levels declined relative to pre-pregnancy levels, and that many women relied on non-medical sources of information about exercise during pregnancy [51]. Population-level analyses further suggested that built environment characteristics, particularly neighborhood walkability, were associated with both higher maternal physical activity levels and more favorable pregnancy outcomes [52].

Across domains, several studies reported outcomes spanning multiple categories, underscoring the multidimensional and heterogeneous nature of the current evidence base. While walking-based outdoor activity was the most frequently studied exposure modality, environmental context, behavioral patterns, and outcome selection varied considerably across study designs. The characteristics and key findings of all 22 included studies, including statistical significance where reported, are presented in Table 4.

## 4. Discussion

The 22 studies mapped in this review reveal a complex and heterogeneous evidence landscape. To structure the interpretation, the discussion that follows is organized by health outcome domain, with attention to how findings differ across the three exposure categories identified in the Results: structured walking-based interventions, self-reported habitual outdoor activity, and environmental proxy measures. Where available, findings are contextualized against the broader prenatal exercise and environmental health literature.

### 4.1. Metabolic Outcomes and Glycaemic Regulation

Several included studies indicate that regular walking, an accessible and low-cost form of activity, may support improved glycaemic control in pregnant women with gestational diabetes mellitus (GDM) or those at elevated risk [37,38,42,45,47]. In a factorial trial of 200 women with GDM, brisk walking for 20 min per day was associated with lower postprandial glucose, glycated haemoglobin, blood lipids, and inflammatory markers, alongside approximately halved maternal and neonatal complications [38]. Consistent patterns were reported in a longitudinal study with objective step monitoring, in which achieving at least 6000 steps/day was linked to better glycaemic control [45], and in an eight-week walking programme targeting a similar step volume that reduced fasting glucose and glycated haemoglobin in women with overweight [47]. The WINGS project reported with substantially lower risk of adverse neonatal outcomes among women with GDM, whereas sedentary behavior was associated with markedly higher risk; notably, only 10% of participants met recommended physical activity levels, underscoring the gap between guidelines and actual behavior [42].

These findings are broadly consistent with the wider prenatal exercise literature. A meta-analysis of 20 randomized trials (*n* = 6767) reported that exercise during pregnancy reduced the risk of GDM by 34% (RR = 0.66; 95% CI 0.50–0.86), confirming that physical activity exerts a protective effect on glucose metabolism [4]. However, the majority of trials in that meta-analysis did not distinguish between indoor and outdoor exercise settings, making it difficult to determine whether the outdoor context itself confers additional metabolic advantages. Whether the exercise setting independently contributes to health outcomes remains unresolved.

By contrast, studies relying on self-reported outdoor activity and environmental proxy measures yielded less direct evidence for metabolic endpoints. The prospective cohort by Bjørn Jensen et al. [37] demonstrated that self-reported outdoor physical activity independently predicted serum 25-hydroxyvitamin D concentrations but did not assess glycaemic outcomes. Population-level analyses of neighborhood walkability reported associations with lower GDM rates [52,55], but these indicators capture the supportive environmental context for physical activity rather than actual metabolic response to exercise, a distinction explored further in Section 4.5.

At the same time, not all activity targets appear feasible in real-world settings. A large Canadian trial combining dietary counselling with a walking component aiming for 10,000 steps/day did not achieve its primary outcome despite improvements in dietary quality, with the mean daily step count remaining around 6300 [50]. This finding, together with the step thresholds observed in other included intervention studies [45,47], suggests that approximately 6000 steps per day may represent a more realistic and clinically meaningful target for metabolic benefit than higher goals that prove difficult to sustain during pregnancy.

More broadly, recent trial-quality-focused evidence syntheses urge caution in interpreting these associations. Poprzeczny et al. [56] reported that when analyses were restricted to trials at no or negligible risk of bias, no significant reductions in GDM incidence (RR = 0.60; 95% CI 0.16–2.23) or gestational weight gain (WMD = −0.60 kg; 95% CI −2.17 to 0.98) were demonstrated, whereas inclusion of methodologically weaker trials shifted estimates further from the null. This is particularly relevant for the present review, as most included walking interventions had small samples and did not consistently report intention-to-treat analyses, which may have inflated apparent metabolic effects.

### 4.2. Cardiovascular Function

Cardiovascular outcomes were less frequently assessed than metabolic or mental health measures across the included studies. All three studies that examined cardiovascular endpoints employed structured intervention designs; none of the studies that relied on self-reported habitual outdoor activity or environmental proxy measures reported cardiovascular outcomes [39,46,53]. This concentration of evidence within a single exposure category limits the scope of conclusions that can be drawn about the relationship between outdoor environments and maternal cardiovascular health.

Among the intervention studies, walking-based programs were associated with favorable changes in maternal blood pressure and related parameters. In a small randomized trial among pregnant women with anemia, Nordic walking with poles over three weeks reduced heart rate and blood pressure and was associated with improved fetal cardiotocography parameters, suggesting better fetal adaptation to mild hypoxic stress [39]. In another randomized trial of women at increased risk of hypertensive disorders, a structured walking program of 20–30 min four times per week from 14 to 34 weeks of gestation significantly reduced the incidence of gestational hypertension and was associated with fewer cases of preeclampsia [46]. Although both trials reported statistically significant results, their relatively small sample sizes (*n* = 110 and *n* = 72, respectively) and absence of blinding warrant caution in generalizing these findings.

These results are broadly consistent with external evidence. A recent meta-analysis of randomised trials reported that physical activity during pregnancy was associated with a 56% lower risk of gestational hypertensive disorders (RR = 0.44; 95% CI 0.30–0.66) and modest reductions in both systolic and diastolic blood pressure, although the effect on diagnosed preeclampsia specifically was not statistically significant (RR = 0.81; 95% CI 0.59–1.11) [57]. This outcome-specific variability is reflected in the present review, which included trials that also suggested a trend toward lower preeclampsia incidence that did not reach statistical significance.

Beyond blood pressure regulation, differences across activity modalities may be relevant to cardiovascular and autonomic physiology. A cohort comparison between women participating in structured prenatal yoga and those undertaking moderate-intensity walking indicated that yoga was associated with greater parasympathetic activation, lower sympathetic stress response, and faster recovery following an acute psychological stress task [53]. These findings suggest that walking-based activity primarily influences hemodynamic parameters, whereas yoga may confer additional benefits through autonomic regulation and stress recovery pathways. However, the study was non-randomized, had a small sample (*n* = 69), and did not control for self-selection into activity modalities. The absence of studies examining cardiovascular outcomes beyond structured intervention designs limits the scope of inference in this domain.

### 4.3. Mental Health and Psychosocial Outcomes

One of the most consistent signals across the included literature concerns the relationship between contact with nature, outdoor activity, and maternal mental health [3,27,32,41,54]. Importantly, and in contrast to the metabolic and cardiovascular domains, positive associations were reported across all three exposure categories, structured interventions [3], self-reported outdoor activity [27,32], and environmental proxy measures [41,54], suggesting that both active engagement in physical activity and passive environmental exposure may be relevant to maternal psychological well-being.

Among environmental proxy measures, the largest included study, a cross-sectional analysis within the Born in Bradford cohort (*n* = 7547), found that women living in greener areas had 18–23% lower odds of depressive symptoms, with the association being stronger among women with lower socioeconomic status and among those who were physically active [41]. Although physical activity partially mediated this association, it explained only 5–8% of the total effect, implying additional pathways such as psychological restoration, social interaction, or reduced air pollution exposure. A recent longitudinal Canadian cohort study (*n* = 10,866) reinforced these findings, reporting that higher residential greenness and closer proximity to blue spaces were associated with fewer perinatal depressive symptoms, with the strongest effects observed during the prenatal period [58]. Together, these studies suggest that residential exposure to natural environments may exert protective effects on maternal mental health through pathways that extend beyond facilitating physical activity alone.

However, not all evidence supports a straightforward relationship between greenness and mental health. An observational cohort from New Zealand found no significant association between residential greenness and antenatal depression after full covariate adjustment [43], and a separate analysis from the same study population reported that green space was not associated with meeting physical activity recommendations after accounting for neighborhood preference [43]. These contrasting findings suggest that residential proximity to green space is not equivalent to actual use, and that the quality, safety, and accessibility of these spaces may matter more than their mere presence. The role of environmental quality and accessibility is examined in greater detail in Section 4.5.

Among self-reported outdoor activity studies, the most striking finding came from the pandemic context. In a large cross-sectional survey of over 8300 pregnant women during COVID-19, outdoor physical activity was among the most commonly used coping strategies and was associated with approximately 29–38% lower prevalence of moderate-to-severe depressive symptoms [27]. Complementary qualitative evidence indicated that cycling during pregnancy was associated with perceived autonomy and well-being, although continuation depended on safety perceptions and social support [32]. A recent study from Ghana further extended this evidence to a low- and middle-income setting, reporting that maternal use of green spaces for recreation and exercise was associated with lower perceived stress, anxiety, and depression [54], supporting the cross-cultural relevance of outdoor environments for maternal mental health.

Among structured intervention studies, a small pilot walking program among clinically depressed pregnant women (*n* = 18) demonstrated feasibility, high completion rates, and a reduction in depressive and anxiety symptoms [3]. Although the absence of a control group limits causal inference, this study provides preliminary support for walking as a low-cost intervention for prenatal depression. A recent systematic review of nature-based interventions for perinatal mental health identified only three studies (*n* = 68 total) and concluded that current evidence is insufficient to draw firm conclusions, while clearly indicating the need for more rigorous trial designs [59]. This assessment is consistent with the broader pattern observed in the present review: while the direction of evidence is largely favorable, the evidence base for structured outdoor interventions targeting prenatal mental health remains at an early stage of development.

Evidence from the broader literature provides additional context. A meta-analysis of 15 randomized trials reported moderate improvements in mental health with urban green exercise compared with control conditions [60], and a separate meta-analysis reported consistent associations between higher prenatal physical activity and improved postpartum mental health [61]. Although neither review was specific to pregnant populations or to outdoor settings, these findings support the plausibility of outdoor physical activity as a contributor to maternal psychological well-being. However, causal inference remains limited in this domain.

### 4.4. Pregnancy and Birth Outcomes

Evidence on pregnancy and birth outcomes was more heterogeneous than that for metabolic and mental health outcomes, with studies reporting protective associations [38,49,51,52], null findings [50], and non-linear relationships [40,48].

Among structured walking-based interventions, a randomized trial of walking four times weekly from 34 weeks until delivery reported improved cervical readiness, higher rates of spontaneous labor onset, and reduced cesarean and induction rates without adverse neonatal effects [49], suggesting that late-pregnancy walking may influence cervical ripening and labor progression. As noted in the metabolic outcomes discussion above, the factorial trial by Bo et al. [38] also reported approximately halved maternal and neonatal complications with daily walking among women with GDM, and the WINGS project demonstrated that recreational walking was associated with substantially lower risk of adverse neonatal outcomes in this high-risk population [42]. These findings suggest that the metabolic improvements associated with walking may translate into better birth outcomes, particularly in women with gestational diabetes. However, the BHIP trial [50] again illustrates the challenge of achieving ambitious activity targets in real-world settings.

Self-reported outdoor activity studies yielded more heterogeneous results, particularly regarding preterm birth. A large case-control study in China (*n* = 6656) reported a dose-dependent association between outdoor exercise frequency and lower odds of preterm birth, with reductions of 20–32% across increasing activity categories; however, the study also identified a possible non-linear pattern at very high volumes of activity, although this subgroup was small and estimates imprecise [48]. A cross-sectional study from Poland reported that physically active pregnant women had approximately 30% lower risk of cesarean delivery, although the study relied on self-reported data [51]. These findings are broadly consistent with a meta-analysis of 57 studies, which confirmed that physical activity during pregnancy does not increase the risk of preterm birth (RR = 0.96; 95% CI 0.77–1.21) [57], providing reassurance regarding the safety of continued outdoor activity throughout pregnancy.

A distinct category of outdoor exposure concerns high-altitude environments. In a survey of 459 women who traveled to or resided above 2440 m during pregnancy, high-altitude exposure combined with outdoor activity was associated with increased odds of preterm labor and neonatal oxygen use, while other complications were not markedly more frequent [40]. By contrast, a case report of a Sherpa woman at 31–32 weeks of gestation completing an ascent to Everest Base Camp without apparent complications suggests that, in populations adapted to high altitude, extreme activity may be feasible [44]. These data support individualized guidance that accounts for baseline acclimatization and population-specific adaptation.

At the population level, environmental proxy measures linked supportive built environments to favorable pregnancy outcomes [52], although these associations are examined in the environmental context discussion below. The broader methodological concerns raised by trial-quality–focused syntheses [56] apply with particular force to this outcome domain, where the evidence is most heterogeneous and where small sample sizes and observational designs are most prevalent.

### 4.5. Environmental Context: Green Space and Conditions That Support Walking

Beyond the direct effects of activity, several studies examined environmental characteristics that may shape opportunities for outdoor movement [41,43,52,54]. This domain is conceptually distinct from the preceding outcome-focused sections, as it addresses the contextual conditions under which outdoor activity occurs rather than its physiological or psychological consequences.

The strongest population-level evidence came from an analysis linking a national index of neighbourhood walkability to approximately 3.28 million U.S. birth records, which reported that living in areas more supportive of walking was associated with higher maternal physical activity and lower risks of preterm birth, low birth weight, gestational diabetes, and gestational hypertension, although associations with excessive gestational weight gain were less clear [52]. A cross-sectional analysis of over 109,000 births in New York City corroborated these findings, reporting that women in the most walkable neighborhoods had a 19% lower risk of gestational diabetes compared with those in the least walkable areas [55]. These converging results suggest that built environments designed to support pedestrian activity may facilitate health-promoting behaviors during pregnancy, although walkability indices are area-level measures that do not capture individual behavior, and self-selection remains a plausible alternative explanation.

Evidence from the broader literature reinforces the relevance of the built environment for prenatal physical activity. A large multisite cohort (*n* = 8362) found that greater availability of gyms and recreation areas, and higher walkability, were each associated with meeting physical activity guidelines during pregnancy, with these associations strengthening across trimesters [62]. A prospective cohort study reported that high neighborhood walkability was associated with more than twofold higher odds of adequate total physical activity during pregnancy, whereas greater distance from parks significantly reduced recreational activity [29]. These findings underscore that functional access, encompassing pedestrian infrastructure, proximity to destinations, and connectivity, may be more important for promoting prenatal physical activity than the mere presence of green space.

However, the evidence does not uniformly support the simple interpretation that greater green space availability directly translates into higher physical activity levels. As noted in the mental health discussion above, the Growing Up in New Zealand cohort found no significant associations between neighborhood green space and either physical activity or antenatal depression after full covariate adjustment [43]. These null findings suggest that residential greenness and walkability may operate through distinct pathways: walkability captures functional access, whereas greenness reflects vegetation density, which may influence health through restorative, air quality, or aesthetic pathways rather than by directly promoting physical activity [18].

At the same time, residential greenness does appear to be associated with certain pregnancy outcomes at the population level. Meta-analyses of observational studies have reported that higher residential greenness was associated with modestly higher birth weight and reduced odds of low birth weight and small for gestational age, although associations with preterm birth, gestational diabetes, and gestational hypertension were not consistently significant [19,63,64]. While these findings suggest potential population-level benefits, effect sizes are modest, between-study heterogeneity is substantial, and the mechanisms remain unclear.

Qualitative evidence from this review highlights social and structural barriers that may attenuate the effects of the environment on outdoor activity during pregnancy. Pregnant British Pakistani women in deprived UK areas reported safety concerns related to crime, cultural constraints, and embarrassment about exercising outdoors, poor park quality, and a lack of female-only facilities, all of which reduced engagement despite physical proximity to green spaces [28]. Women who cycled during pregnancy reported that continuation depended on perceived road safety and social support rather than infrastructure availability alone [32]. Conversely, the Ghana study [54] indicated that where barriers are lower, environmental engagement may translate more readily into health benefits. These contrasting findings reinforce the importance of considering not only the physical availability of outdoor spaces but also their perceived safety, cultural acceptability, and quality.

Both walkability and residential greenness may contribute to maternal health, but through partially distinct pathways. Walkability appears to influence health primarily by facilitating physical activity, whereas greenness may operate through additional mechanisms, including stress reduction, improved air quality, and psychological restoration.

### 4.6. Methodological Heterogeneity and Limitations of the Evidence

A central challenge in interpreting the findings of this scoping review is the variability in how outdoor exposure was defined, operationalised, and measured across the 22 included studies. Three fundamentally different approaches were employed (structured walking-based interventions, self-reported habitual outdoor activity, and environmental proxy measures), each capturing related but non-equivalent dimensions of outdoor exposure. This heterogeneity means, for example, that the metabolic benefits observed in a structured walking trial among women with gestational diabetes cannot be directly compared with the mental health associations reported in a population-level greenness analysis. This conceptual and methodological heterogeneity limits direct comparability across studies and reduces the internal consistency of the evidence base, thereby constraining the strength of overall inferences. The majority of included studies employed observational designs, making it unclear, for instance, whether pregnant women with better mental health preferentially seek out green spaces, or whether exposure to greenness itself reduces depressive symptoms. The interventional studies were of short duration, ranging from 21 days [39] to approximately eight weeks [47], restricting conclusions regarding sustained effects throughout pregnancy.

Within each exposure category, specific methodological concerns warrant consideration. Among the intervention studies, most had small sample sizes, typically fewer than 110 participants, and dropout rates exceeding 20% were reported in at least one trial [39]. Several studies recruited highly specific populations, such as pregnant women with anaemia residing at a health resort [39] or physically active mountaineering women recruited via online platforms [40], introducing selection bias that limits the applicability of findings to the broader obstetric population. None of the trials blinded participants, and inconsistent reporting of intention-to-treat analyses raises the possibility that post-randomisation exclusions may have contributed to more favourable outcomes in the intervention groups. These features increase the risk of selection and attrition bias and limit the external validity of intervention findings. Although the included intervention studies were selected on the basis that physical activity occurred in outdoor settings, none of them treated the outdoor environment as an independent variable or compared outcomes with an equivalent indoor condition, making it impossible to determine whether the outdoor context conferred additional benefits beyond the exercise stimulus itself.

Studies relying on self-reported outdoor activity used retrospective questionnaires of varying psychometric quality, including the short-form IPAQ [43] and the GPPAQ [41], neither of which was designed to distinguish between indoor and outdoor activity settings, increasing the risk of exposure misclassification. This may lead to attenuation or inflation of observed associations and reduces the precision of effect estimates. Recall bias is of particular concern where pregnancies occurred up to 25 years prior to data collection [40]. Such bias may compromise the accuracy of reported exposure and outcome relationships, thereby affecting internal validity. Only three studies employed objective monitoring [3,42,45], and these were limited by small samples. Outcome ascertainment also varied across the evidence base, with some studies relying on self-reported psychological symptoms [21,41,54], coping behaviors [27], or physical activity patterns [32,40,43,51], whereas others used clinical [38,46,47,49,52], physiological [39,42,45,53], or registry-derived outcomes [37,52], further complicating cross-study comparison.

Environmental proxy studies introduced distinct challenges. Area-level measures such as NDVI and walkability indices do not reflect individual exposure and cannot distinguish between a private garden and a publicly accessible park [41]. This introduces exposure misclassification and limits causal interpretation at the individual level. Hospital-based sampling in one study excluded mothers without regular healthcare access, restricting generalisability to peri-urban and rural populations [54]. Residual confounding was not uniformly addressed; notably, Nichani et al. [43] demonstrated that the association between green space and physical activity disappeared entirely once neighbourhood lifestyle preference was included as a covariate, underscoring the importance of controlling for residential self-selection in environmental epidemiology of pregnancy. Reliance on self-reported outcomes without medical record verification and cultural stigma surrounding mental illness may have further compromised the accuracy of outcome measurement, particularly in studies assessing maternal psychological well-being [54]. This may lead to systematic under- or overestimation of associations, particularly in socially sensitive domains such as mental health. The timing of exposure measurement also varied considerably, with assessments ranging from approximately 25 weeks to after 34 weeks of gestation and limited repeated measurement across pregnancy. The geographical and cultural diversity of the included studies (conducted across 15 countries spanning six continents), while a strength in terms of breadth, also introduces variability in healthcare systems, cultural norms regarding physical activity during pregnancy, and climatic conditions that may modify the observed associations. This variability limits the generalizability of findings across populations and healthcare contexts.

Several limitations of the review itself should be acknowledged. The search was restricted to English-language publications, and grey literature was not systematically included. The heterogeneity in exposure definitions and outcome measures precluded meta-analysis, and critical appraisal was conducted for contextual insight rather than as a basis for study exclusion, consistent with scoping review methodology.

Collectively, these issues weaken internal validity within individual studies, limit causal inference across the evidence base, and reduce the generalisability of findings across obstetric populations, healthcare settings, and sociocultural contexts. Taken together, these limitations indicate that while 17 of 22 studies reported at least one positive association, the evidence remains methodologically uneven across exposure categories. Structured walking interventions provided the most direct evidence of metabolic and cardiovascular benefits, but were generally conducted in small, clinically selected samples. Self-reported activity studies and environmental proxy studies broadened the evidence base, but were limited by indirect or non-specific exposure assessment. Future research should combine objective physical activity monitoring with geo-located environmental exposure assessment, employ repeated measurement across pregnancy, and design trials comparing indoor and outdoor exercise conditions.

## 5. Conclusions

This scoping review identified 22 studies investigating outdoor physical activity and nature-based environmental exposure during pregnancy across a wide range of study designs, populations, settings, and outcome domains. Taken together, the available evidence suggests that walking-based outdoor activity and supportive environmental conditions may be associated with beneficial metabolic, cardiovascular, mental health, and selected pregnancy-related outcomes. The most consistent signals emerged for metabolic outcomes and maternal mental health, whereas evidence for pregnancy and birth outcomes remained more heterogeneous.

At the same time, the current evidence base remains methodologically diverse. Considerable variation in exposure definitions, measurement strategies, timing of assessment across gestation, and overall study design limits direct comparison between studies and reduces confidence in causal interpretation. Structured intervention studies offer the most direct evidence of potential benefit, whereas studies based on self-reported activity or environmental proxy indicators capture broader but less precise dimensions of outdoor exposure. These pathways may overlap through increased movement, stress reduction, or improved opportunities for engagement, but they should not be considered interchangeable.

Future research should therefore move beyond broad exposure categories and adopt more precise and consistent methodological approaches. Priority should be given to clearly identifying whether physical activity is undertaken outdoors, using standardized exposure metrics, incorporating repeated assessments across pregnancy, and conducting adequately powered trials with robust analytical methods. Greater inclusion of socioeconomically and culturally diverse populations will also be essential to improve generalizability and equity in evidence-based recommendations. Strengthening methodological rigor in this field will be necessary to determine whether outdoor environments confer health benefits beyond those attributable to physical activity alone and to clarify how such benefits may be translated into clinical guidance and public health policy.

## Figures and Tables

**Table 1:** Search Strategies Used Across Databases.

Database	Search Strategy
PubMed	(pregnan*) AND (“physical activit*” OR “outdoor physical activit*” OR exercis* OR walk* OR yoga OR “green exercise”) AND (outdoor* OR “green space*” OR greenspace* OR greenness OR “blue space*” OR “nature-based”)
Scopus	TITLE-ABS-KEY ((pregnan*) AND (“physical activit*” OR “outdoor physical activit*” OR exercis* OR walk* OR yoga OR “green exercise”) AND (outdoor* OR “green space*” OR greenspace* OR greenness OR “blue space*” OR “nature-based”)) AND PUBYEAR > 2012 AND (LIMIT-TO (LANGUAGE, “English”))
Web of Science	(pregnan*) AND (“physical activit*” OR “outdoor physical activit*” OR exercis* OR walk* OR yoga OR “green exercise”) AND (outdoor* OR “green space*” OR greenspace* OR greenness OR “blue space*” OR “nature-based”)
Google Scholar (Supplementary)	(pregnancy) AND (“physical activity” OR “physical activities” OR “outdoor physical activity” OR “outdoor physical activities” OR exercise OR walking OR yoga OR “green exercise”) AND (outdoor OR “green space” OR greenspace OR greenness OR “blue space” OR “nature-based”)

**Table 2:** Inclusion and exclusion criteria based on the PICOS framework.

PICOS Element	Inclusion Criteria	Exclusion Criteria
Population (P)	Pregnant women at any gestational age.	Women who are not pregnant; studies without data collected during pregnancy.
Intervention (I)	Outdoor physical activity, including walking, running, cycling, swimming in natural environments, green or blue space exposure involving physical activity, and structured outdoor exercise programs.	Physical activity performed exclusively in indoor settings or without exposure to outdoor or natural environments.
Comparison (C)	Indoor physical activity, usual care, low physical activity, or no physical activity, when applicable.	Not applicable.
Outcomes (O)	Physical and mental health outcomes, including metabolic health (e.g., gestational diabetes, glycemic control, gestational weight gain), mental health (e.g., stress, anxiety, depression), and pregnancy or birth outcomes (e.g., preterm birth, mode of delivery, macrosomia).	Studies that do not report health-related outcomes.
Study Design (S)	Original peer-reviewed research studies of any methodological design, including randomized controlled trials, observational studies (cohort, case–control, and cross-sectional), quasi-experimental studies, pilot studies, case series, and qualitative studies.	Secondary research (systematic reviews, meta-analyses, and scoping reviews), non-original publications (editorials, commentaries, letters), conference abstracts without full-text availability, study protocols, dissertations, theses, guidelines, expert opinions, and grey literature.

**Table 3:** Methodological quality assessment of included studies.

Study (Author, Year)	Design	Tool	Score	Level	Comments
Bjørn Jensen et al., 2013 [37]	Cohort study	NOS	8/9	High	Large prospective cohort; self-reported physical activity.
Bo et al., 2014 [38]	RCT	PEDro	7/10	High	No blinding of participants and therapists.
Vladimirov et al., 2015 [39]	RCT	PEDro	6/10	High	No blinding; small final sample size.
Keyes et al., 2016 [40]	Cross-sectional	JBI	6/9	Moderate	Self-reported data; recall bias; selected active population.
McEachan et al., 2016 [41]	Cross-sectional	JBI	7/9	High	Self-reported depressive symptoms.
Anjana et al., 2016 [42]	Quasi-experimental	JBI	7/9	High	No randomization; combined lifestyle intervention; partial objective PA measurement.
Nichani et al., 2016 [43]	Cross-sectional	JBI	7/9	High	Self-reported physical activity.
Bennett, 2017 [32]	Qualitative	CASP	9/10	High	Small sample size; no data saturation.
Davenport et al., 2018 [44]	Case study	JBI	6/9	Moderate	No comparator; limited generalizability.
Hayashi et al., 2018 [45]	Cohort study	NOS	6/9	Moderate	Small sample; single-center; no control group.
Khoram et al., 2019 [46]	RCT	PEDro	7/10	High	No blinding of participants and therapists.
Battle et al., 2023 [3]	Pilot trial	JBI	6/9	Moderate	No control group; non-randomized; small sample; feasibility focus.
Ghani, 2021 [47]	Quasi-experimental	JBI	7/9	High	No blinding; single-center study.
Cai et al., 2021 [48]	Case–control	NOS	8/9	High	Self-reported PA; potential recall bias.
Shojaei et al., 2021 [49]	RCT	PEDro	7/10	High	No blinding of participants and assessors.
Atkinson et al., 2022 [50]	RCT	PEDro	8/10	High	Open-label design; PA goal not fully achieved.
Badon et al., 2022 [27]	Cross-sectional	JBI	7/9	High	Self-reported coping strategies and outcomes.
Misan et al., 2022 [51]	Cross-sectional	JBI	6/9	Moderate	Self-reported PA; single-center study.
Conway & Menclova, 2023 [52]	Cross-sectional	JBI	7/9	High	Potential residual confounding.
Iqbal et al., 2024 [28]	Qualitative	CASP	9/10	High	Small sample; limited generalizability.
Lučovnik et al., 2024 [53]	Cohort study	NOS	7/9	High	Non-randomized; small sample; potential confounding.
Boakye et al., 2025 [54]	Cross-sectional	JBI	7/9	High	Self-reported green space exposure and mental health outcomes.

Note: PEDro scores (items 2–11; range 0–10) were classified a priori as high (≥6), moderate (4–5), or low (≤3). NOS scores (0–9 stars) were classified as high (≥7), moderate (4–6), or low (≤3). For JBI checklists, high quality indicated ≥70% of applicable criteria met, moderate 50–69%, and low <50%. CASP scores (0–10) were classified as high (≥8), moderate (6–7), or low (≤5). Scores were assigned independently by two reviewers, and disagreements were resolved by consensus.

**Table 4:** Characteristics of included studies examining outdoor physical activity during pregnancy.

Author/Year	Country	Design	Sample	Gestation	Outdoor Activity	Exposure Parameters	Outcome Domain	Key Findings
Bjørn Jensen et al. (2013) [37]	Denmark	Prospective cohort	N = 1494	∼25 weeks	Outdoor PA (self-reported)	Minutes/week	Metabolic	Outdoor PA independently predicted serum 25(OH)D levels (p=0.008; final model $R^{2}=0.401$, p<0.001).
Bo et al. (2014) [38]	Italy	Factorial RCT	N = 200 (GDM)	24–26 weeks	Brisk walking	20 min/day, daily	Metabolic; Birth	Reduced postprandial glucose (p<0.001), HbA1c (p<0.001), TG (p=0.02), CRP (p<0.001), and maternal or neonatal complications (OR = 0.50; 95% CI 0.28–0.89; p=0.02).
Vladimirov et al. (2015) [39]	Italy/ Ukraine	RCT	N = 110 (IDA)	20–27 weeks	Medical pole walking	25–30 min/day for 21 days	Cardiovascular; Fetal	Reduced maternal HR and BP (p<0.05); improved fetal HR parameters (p<0.05).
Keyes et al. (2016) [40]	USA	Cross-sectional	N = 459	All trimesters	Outdoor PA; high-altitude exposure	Self-reported	Maternal; Perinatal	Higher odds of preterm labor (OR = 2.3; 95% CI 0.97–5.4; p=0.05) and neonatal oxygen use (OR = 2.34; 95% CI 1.04–5.26; p<0.05); NICU admission not significant.
McEachan et al. (2016) [41]	UK	Cross-sectional	N = 7547	∼28 weeks	Residential greenness (NDVI)	Chronic exposure	Mental	Higher greenness associated with lower depressive symptoms (OR = 0.82; 95% CI 0.69–0.98; p<0.05), stronger among women with lower SES (OR = 0.74; p<0.05) and higher PA (OR = 0.63; p<0.05).
Anjana et al. (2016) [42]	India	Pre–post intervention	N = 151 (GDM)	<28 weeks	Recreational walking	Step count increased; ∼12 weeks	Metabolic; Neonatal	Improved glycaemic control and neonatal outcomes (fasting and postprandial glucose, p<0.001; sedentary behaviour OR = 3.8, 95% CI 1.2–12.2, p=0.02; walking protective OR = 0.3, 95% CI 0.07–1.0, p=0.04).
Nichani et al. (2016) [43]	New Zealand	Prospective cohort	N = 6772	Across pregnancy	General PA; green space exposure	≥150 min/week guideline	Behavioral	Green space not associated with meeting PA recommendations after adjustment (OR = 1.16; 95% CI 0.99–1.36; p>0.05).
Bennett (2017) [32]	UK	Qualitative	N = 14	All trimesters	Cycle commuting	Self-reported	Mental; Social	Cycling associated with wellbeing and autonomy; influenced by safety perceptions and social context.
Davenport et al. (2018) [44]	Nepal	Case report	N = 1	31–32 weeks	High-altitude trekking	250–300 min/day	Maternal; Fetal	No apparent maternal or neonatal complications in a single case.
Hayashi et al. (2018) [45]	Japan	Longitudinal cohort	N = 24 analysed	Mid-late pregnancy	Daily walking	7–12 weeks	Metabolic	≥6000 steps/day associated with lower glucose levels (r=−0.603; p=0.013).
Khoram et al. (2019) [46]	Iran	RCT	N = 72	14–34 weeks	Walking	20–30 min, 4×/week	Cardiovascular	Reduced systolic and diastolic BP and hypertensive disorders (p<0.05).
Battle et al. (2023) [3]	USA	Pilot trial	N = 18	12–26 weeks	Walking	10 weeks, daily goals	Mental	Feasible and acceptable (83% completion); reduced depressive and anxiety symptoms (depressive symptoms p<0.05; anxiety not significant).
Ghani (2021) [47]	Egypt	Quasi-experimental	N = 100	Second trimester	Walking program	Self-reported	Metabolic	Lower fasting glucose (p=0.01) and HbA1c (p=0.05); GDM incidence not significant (p=0.16).
Cai et al. (2021) [48]	China	Case-control	N = 6656	All trimesters	Outdoor PA	Self-reported	Perinatal	Lower PTB odds at 1–2/week (aOR = 0.80; 95% CI 0.68–0.92), 3–4/week (aOR = 0.70; 95% CI 0.60–0.82), and ≥5/week (aOR = 0.68; 95% CI 0.59–0.78); U-shaped pattern by duration.
Shojaei et al. (2021) [49]	Iran	RCT	N = 100	≥34 weeks	Walking	40 min, 4×/week	Labor; Birth	Improved Bishop score and spontaneous labor (p<0.001); reduced induction (p=0.05) and caesarean delivery (p<0.001).
Atkinson et al. (2022) [50]	Canada	RCT	N = 241	12 weeks to delivery	Walking + nutrition	3–4×/week	Metabolic; Birth	Improved diet quality (p<0.001); no effect on GWG or birth outcomes (GWG OR = 1.51; 95% CI 0.81–2.80; p>0.05).
Badon et al. (2022) [27]	USA	Cross-sectional	N = 8320	All trimesters	Outdoor PA (coping)	Self-reported	Mental	Outdoor PA as coping associated with lower depressive and anxiety symptoms (depression PR = 0.63; 95% CI 0.55–0.73; anxiety PR = 0.66; 95% CI 0.57–0.76; p<0.05).
Misan et al. (2022) [51]	Poland	Cross-sectional	N = 247	All trimesters	Walking predominant	Self-reported	Behavioral; Birth	PA associated with lower caesarean risk (OR = 2.05; 95% CI 1.15–3.65; p<0.05); activity declined vs. pre-pregnancy (p<0.001).
Conway & Menclova (2023) [52]	USA	Population-based	∼3.28 M births	≥26 weeks	Walkability index	Environmental exposure	PA; Birth	Higher walkability associated with higher PA (+39.31 min/week per 10-point WI increase), and lower PTB, LBW, GDM, and gestational hypertension (p<0.05).
Iqbal et al. (2024) [28]	UK	Qualitative	N = 21	26–28 weeks	Walking; environment	Perceptions	Behavioral; Mental	Safety and cultural barriers influenced outdoor activity.
Lučovnik et al. (2024) [53]	Slovenia	Prospective cohort	N = 69	Across pregnancy	Yoga vs. walking	Weekly sessions	Cardiorespiratory	Yoga showed greater parasympathetic activity and faster stress recovery vs. walking (F=31.26; p<0.001).
Boakye et al. (2025) [54]	Ghana	Cross-sectional	N = 420	Postnatal	Green space exposure	Self-reported	Maternal mental health	Green space use was linked to lower stress (β=−0.118; p=0.016); belief in benefits was linked to higher anxiety (β=0.117; p=0.017) and depression (β=0.164; p=0.001).

Note: PA = physical activity; RCT = randomized controlled trial; Factorial RCT = randomized controlled trial with factorial design; Pre–post intervention = non-randomized intervention with before–after comparison; GDM = gestational diabetes mellitus; BP = blood pressure; HR = heart rate; NDVI = normalized difference vegetation index; SES = socioeconomic status; PTB = preterm birth; LBW = low birth weight; GWG = gestational weight gain; IDA = iron deficiency anemia.

## Data Availability

No new primary data were created in this study. All data supporting the findings of this review are derived from previously published articles, which are cited in the reference list. Extracted data and appraisal tables are available from the corresponding author upon reasonable request.

## Abbreviations

The following abbreviations are used in this manuscript:

BP	Blood pressure
CRP	C-reactive protein
EPDS	Edinburgh Postnatal Depression Scale
GDM	Gestational diabetes mellitus
GWG	Gestational weight gain
HbA1c	Glycated hemoglobin
HR	Heart rate
LBW	Low birth weight
NDVI	Normalized difference vegetation index
PA	Physical activity
PTB	Preterm birth
SES	Socioeconomic status
WHO	World Health Organization
